# DECENT: A sociotechnical approach for developing mobile health apps in underserved settings

**DOI:** 10.1177/20552076231203595

**Published:** 2023-09-29

**Authors:** Tochukwu Ikwunne, Lucy Hederman, P. J. Wall

**Affiliations:** 1 ADAPT Centre, School of Computer Science and Statistics, 8809 Trinity College Dublin, Dublin, Ireland

**Keywords:** Mobile health, community health workers, user engagement, user-centred design, Sierra Leone, sociotechnical

## Abstract

**Objective:**

Despite the fact that user engagement is critical to the efficacy of mobile health (mHealth) interventions in the Global South, many of these interventions lack user engagement features. This is because sociotechnical aspects of such initiatives are frequently ignored during the design, development, and implementation stages. This research highlighted the importance of considering sociotechnical factors when developing mHealth apps. The intended users for the mHealth technologies in this study are care professionals.

**Materials and Methods:**

Five semi-structured interviews and a pilot interview were conducted to identify user engagement facilitators and barriers. The interview data were analysed using NVivo. The Capability, Opportunity, Motivation – Behaviour (COM-B) model is then used to map the facilitators and barriers to mHealth app engagement, allowing researchers to better understand how users engage/disengage with mHealth apps.

**Results and Discussion:**

Capability facilitators included features that assist users in learning more about the app (e.g. a user manual and statistical data) as well as features that assist users in developing a routine. The lack of app skills and cognitive overload limit capability. While social connectedness and offline functionality were identified as facilitators of user engagement, non-user-friendly design and cultural dimensions were identified as barriers. Early user engagement and rewards were identified as motivational facilitators that influence user engagement. Furthermore, perceived non-utility and a lack of encouragement were identified as motivational barriers to engagement.

**Conclusion:**

Several factors were discovered across all COM-B model components that could be used to develop more engaging mHealth apps. Adopting a techno-centric approach that ignores sociotechnical factors can reduce user engagement. The design process engagement enhancement system (DECENT) framework was proposed based on the findings.

## Introduction

According to,^1^ ‘*The global South refers broadly to a grouping of countries and people that experience economic marginalisation within the global system and have elements of a shared history of colonisation and exploitation… The global North refers to countries and people traditionally referred to as ‘the West’, who tend to be foreign aid donors to the South’ (p.151).* It is broadly accepted that the ubiquity of mobile phones in the Global South is particularly apparent in West Africa, with 176 million subscribers throughout the subregion as of the year 2017.^1^ However, the design, development, implementation, and use of systems leveraging mobile health (mHealth) have proven to be problematic for a variety of reasons, and a majority of such systems fail to sustain and scale.^2^ There are many reasons put forward for this, for example, Machado et al.^3^ indicated that despite the claimed significance of user engagement for efficacy of mHealth systems, many such interventions frequently do not include user-engaging attributes. User-engaging attributes are strategies that facilitate end-user engagement with mHealth. Cultural issues that transcend and influence the design, development, evaluation, and use of interactive technologies should be explored. Nevertheless, when thinking about a culture mediated by technologies, considering and dealing with cultural concerns have become an even more difficult and crucial challenge.^4^ This paper highlights that sociotechnical factors influencing user engagement in mHealth should be considered as part of the design process of mHealth. The term sociotechnical approaches derive from numerous sources.^5^ Sociotechnical techniques focus on creating interactions from the perspective of the agent rather than the technology.^5^ A sociotechnical approach includes consideration of both social and technical factors based upon a combination of humans and technology.^6^ This research focuses on the origin of the meaning of sociotechnical approaches from Participatory Design, in which users are given the initiative in design to ensure that the tools created better suit their needs and values.^7^ This is because techno-centric approaches alone have been proven to be ineffective.^8^ For example, the literature discusses designers’ assumptions during the design process,^9^ resulting in a lack of user engagement with mHealth systems,^9–11^ as well as a lack of sociotechnical understanding of mHealth design.^12^

### Significance

The research identified a lack of attention to user-engaging attributes as one of the factors to consider in achieving user engagement with mHealth technologies. Other factors include socio-cultural concerns, such as when mHealth apps designed in the Global North are applied in the Global South, where there may be a wide range of social, cultural differences and beliefs. In such instances, localisation of implementation is critical to the success of mHealth solutions.^13^ Shozi et al.^14^ underline this topic as well, claiming that the premise that technology built in the Global North can be simply thrown into the Global South and expected to work is a ‘fallacy’ (p.1). These factors are identified as research gaps. To overcome this research gap, our study argues that the sociotechnical factors that influence user engagement, as well as the socio-cultural contexts of users, should be considered in the design and development of mHealth technologies. We thus propose the following research question: how does consideration of sociotechnical factors in the mHealth design and development process improve user engagement? In order to answer the research question, we examine an mHealth implementation in the Bonthe District of Sierra Leone. Data were collected by semi-structured interviews with mHealth designers and developers to explore their experiences of why users either engage or not with the mHealth application in this case to understand both cultural and techno-centric issues with the use of mHealth technologies. The objective is to identify facilitators and barriers to engagement with mHealth interventions through the lens of the Capability, Opportunity, Motivation – Behaviour (COM-B) model, and to classify these facilitators and barriers as either technical or sociotechnical factors of user engagement. The outcome of the interviews to date indicates that the inclusion of sociotechnical factors during mHealth design and development is a requirement for more effective user engagement with the technology.

## Literature review

This section commences with an examination of the literature on user engagement before moving to examine the body of work relating to mHealth failure and underperformance in the Global South. In particular, attention is paid to the relevance of sociotechnical factors in the design process of mHealth.

### User engagement

There is a wealth of literature on the subject of user engagement with technology. The word ‘user engagement’ refers to the relationship that exists between users and the technology that they utilise. There are several descriptions and definitions of user engagement available, including one by Attfield et al.,^15^ who describes it as ‘the emotional, cognitive, and behavioral connection that exists, at any point in time and possibly over time, between a user and a resource’ (p. 10). User engagement is vital in mHealth, with numerous researchers^16,17^ advocating that the mHealth design process should meet the needs of varied users. Many current mHealth interventions, however, are based on pre-existing healthcare system constructs,^18^ which encourages designers to base their ideas on assumptions that have not been validated with primary user input.^19^ Consequently, the resulting interventions are less effective than those that incorporate end-user needs^18^ and input from key stakeholders such as commercial app businesses and design experts.^16^ Furthermore, according to Korpershoek et al.,^19^ user-centred design is an approach that is informed by the requirements and understanding of a specific end-user group and plays a vital role in fostering user engagement with technology. In addition to this, there is an extensive body of work discussing sociotechnical and socio-cultural factors associated with user engagement,^20–23^ as well as the environment in which the intervention will be used. The work examines the efficacy of prompts to encourage user engagement with digital interventions, as well as the barriers to and facilitators of user engagement with digital mental health interventions. The reviews, however, were not limited to specific study designs, but rather took a much broader look at what factors influence engagement with digital mental health tools. This study focuses on a single research method and technology in the Global South, specifically Sierra Leone mHealth projects, to uncover barriers and facilitators and map them onto a behavioural model to have a deeper understanding of engaging behaviour in a specific context. As stated in the Introduction, sociotechnical factors are used to represent broadly socio-cultural and technical factors. According to Manda and Msosa,^20^ ‘*mHealth comprises multiple sociotechnical arrangements, which, among others, include workers’ information needs, workflow and usability requirements, available technology options, and how best technology can be adapted to suit these needs and requirements’ (p.210).* Thus, there is a need for a better understanding of the complexity of user needs, socio-cultural contexts of users of technologies, and how to incorporate these factors effectively into the design process of mHealth.

### Mobile health failure and underperformance in the Global South

There have been many reasons presented for the high level of mHealth failure and underperformance in the Global South. Kaasbøll and Nhampossa^21^ established that socio-cultural factors have a significant impact on the result of any health information system (HIS) implementation. They explored the socio-cultural issues associated with transfer of HIS between the public health sectors of Mozambique and South Africa. The paper showed that socio-cultural differences between the two countries necessitated considerable adjustment and adaptation to cope with local variations. Similarly, a wide range of papers^24–31^ has argued that successful implementations require a better understanding of sociotechnical practices of user groups and environmental differences because of their significance and impact. The primary conclusions of this research are that, in order to support efficient and effective mobile healthcare, a greater awareness of users’ needs is required, as well as a culture to develop users’ technical skills with necessary and well-matched resources.^24^ Huang et al.^25^ explored how culture influences university technology uptake in two distinct countries. The study discovered that teachers’ perceptions of cultural preferences influenced their constructions and intentions to use technology, albeit the relationship between cultural preferences varied across countries. The huge discrepancies in the values, and uses of app technologies need the development of new methodologies to shine a light on distinctive and integrated technologies concepts across cultures,^27,30^ dimensionalising cultures,^31^ and involvement of relevant stakeholders in the design process of mHealth apps.^28,29^ Additionally, it is recognised that a major reason for failure is unsuitable design as related to the needs and context of use.^32^ Koskinen^33^ posited that global technology carries meanings and structures that may or not fit with local realities and highlights the need for a framework for understanding context that contributes to the understanding of local technology production in under-resourced and developing contexts. Moreover, Wall et al.^3^ observed that the adoption of a techno-centric approach without consideration of sociotechnical issues can negatively affect an mHealth implementation. A further reason for mHealth failure is the use of a top-down approach by implementers.^34^ Such an approach adopts a techno-centric style without allowing users to provide feedback on the technology that they expect to use. Despite the abundance of literature on mHealth and HIS failure and underperformance, there is little discussion about the role of sociotechnical factors in user-centred design processes for mHealth^12,35^ and methods for incorporating users’ socio-cultural contexts into the design process of mHealth technologies. This study emphasised the need of capturing the techno-centric and socio-cultural contexts of mHealth app users during the design and development process.

## Mobile health in Sierra Leone

The research presented in this paper is based on an mHealth case study in the Bonthe District of Sierra Leone, where significant emphasis has been placed on using technology as a key weapon in the fight against disease outbreaks and the promotion of child health. In particular, mobile technologies are viewed by the Ministry of Health and Sanitation (MoHS) in Sierra Leone as an integral component of overall public health strategy with many mHealth initiatives being launched over the past few years by both the MoHS and a variety of non-governmental organisations (NGOs). One example is the use of MOTECH mobile health application, which is a result of collaboration between World Vision Ireland, World Vision Sierra Leone, and Dimagi and Grameen Foundation. These mHealth systems are primarily designed to be used by community health workers (CHWs), who are typically the backbone of healthcare systems in the Global South.^32^ MOTECH is a mobile application developed to be used by CHWs to improve maternal, newborn, and child health across the Bonthe District of Sierra Leone. This system implements the Timed and Targeted Counselling initiative to improve outcomes for maternal and child health and nutrition.

Another example from Sierra Leone is the development of the Mobile Training and Support service (MOTS) to deliver refresher training to CHWs on the topic of vaccines and outbreak response. MOTS is an open-source platform that provides refresher training via an interactive voice response (IVR) system in the participant's preferred language.^36^ It provides mobile training to CHWs via their mobile phones as the basic requirement.^37^ MOTS was initially developed under the Ebola vaccine deployment acceptance and compliance programme and piloted with CHWs to promote the acceptance and uptake of Ebola vaccines.^36,38^ However, the emergence of COVID-19 has introduced another relevant application of MOTS due to the critical need to inform CHWs of health and medical protocols related to the virus without bringing them together for in-person training.

## Methodology

**Figure 1.**
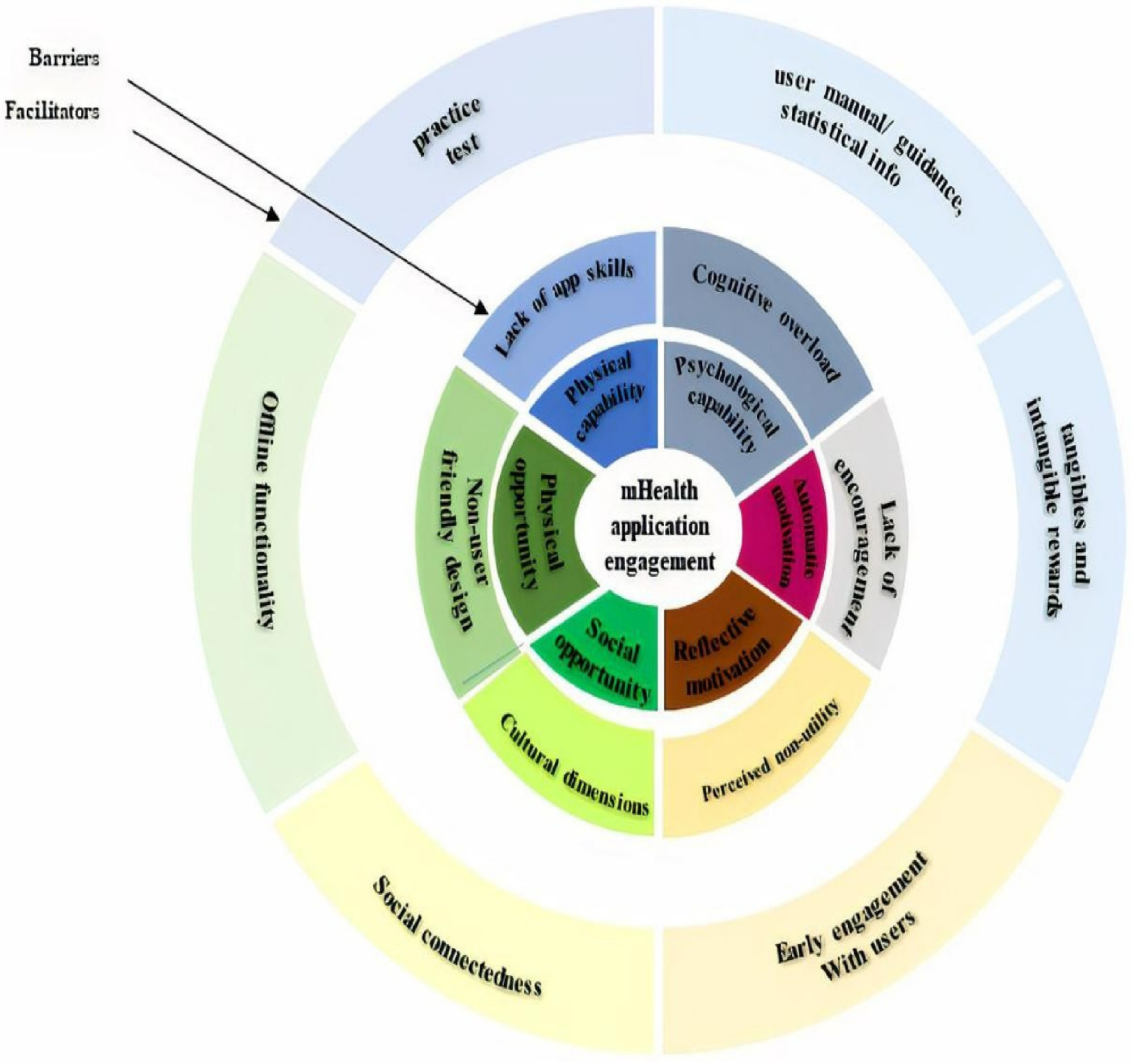
A visual representation of how the facilitators and barriers of mHealth application engagement were mapped onto the components of the COM-B model (adapted from ^39 p.8^).

This paper adopts a qualitative methodological and interpretivist philosophical approach to address the research question. Initially, we rely on five semi-structured interviews and a pilot interview that have been designed to ascertain how designers, developers, and end users of mHealth projects perceive the role of sociotechnical factors as a means to improve user engagement. The COM-B model (Figure 1) is then used to map the facilitators and barriers of mHealth application engagement to understand users’ behaviour in engaging with the mHealth app, with this being discussed further in the results and discussion section.

### Methodological rigour

In order to assess and ensure the robustness of the study, we carefully planned and carried out a series of semi-structured interviews based on the four-dimension criteria (credibility, transferability, dependability, and confirmability), which we adapted from Lincoln and Guba.^40^ The adapted four-dimension criteria used in our study are shown in Table 1.

**Table 1.** Key four-dimension criteria strategies adapted from Lincoln and Guba.^40^

Rigour criteria	Aim	Strategies used in our research to achieve rigour
Credibility	To instil confidence that the results from the viewpoints of the participants are true, credible, and believable.	Two meetings and a pilot interview was used to test the interview protocol. It was ensured that the interview participants had the necessary knowledge and research skills to perform their roles using criterion-based purposive sampling ^41^.
Transferability Dependability	To increase the extent to which results can be generalised to other contexts or settings. The authors (TI, LH, PW) agreed on all of the steps in coding the data and identifying key concepts. The authors (TI, LH, PW) discussed and verified any changes to the coding system to ensure correct and consistent interpretation throughout the analysis.	We used purposive sampling techniques ^41^ to ensure that the participants were representative of the diverse perspectives of participants
Confirmability	To increase the likelihood that the results will be confirmed or corroborated by other researchers.	Each author kept a separate reflexive journal in which they recorded any issues concerning sensitive topics or potential ethical issues that may have affected the data analysis.

The themes within data collected from interviews are identified based on a theoretical or deductive approach since we intend to use the COM-B model to understand users’ behaviour in engaging with the mHealth app. With respect to interview saturation, we assessed the number of new themes added to each interview over time to make sure data saturation was achieved. It shows that the first two interviews yielded the majority of the themes, with themes from subsequent interviews being found less frequently. A total of 12 themes were generated from the interview data. This is consistent with earlier research that assesses the number of interviews required to achieve data saturation for themes, as seen by the decision that six respondents’ interview data was sufficient.^42^ Furthermore, because the respondents are experts in mHealth design and development, the data collected includes information about more than just their own experiences; it also includes information about the experiences of others they know. As a result, the data gathered goes well beyond the ‘*numbers of respondents’.*^43^

### Data collection and methodology

We identified 10 potential participants and conducted five interviews with them (five were unable to participate due to workload availability) and a pilot interview. The overall sample consisted of a group of people who worked in various roles related to the design, development, implementation, and use of mHealth projects in Sierra Leone. World Vision Ireland and World Vision Sierra Leone provided us with information about the key participant information and contact details. Prior to the start of the study, we obtained written informed consent from the subjects, and the study received ethical approval from Trinity College Dublin's School of Computer Science and Statistics Ethics Committee (Reference number: 20200507). A total of five semi-structured interviews were conducted between February and March 2021 with people who have been involved with mHealth projects in Sierra Leone. Each interview with the participants lasted approximately an hour and was conducted in English. The interviews were conducted by one of the authors of this study. We selected a sample of six participants including a pilot interview, from which four are males and two are females, with ages ranging from 25 to 51 years. A snowballing technique was utilised to discover possible interview participants. The participants were contacted by their line managers and asked to participate in the interview process. Those who responded to the request and were then issued the Participant Information Leaflet, which outlined the risks and advantages of the study and gave additional information on the procedures. The participant was contacted by the line manager within 7 days of receiving the information leaflet to confirm that they were still interested in participating in the research. Before the interview began, if the participant consented to continue, he or she was asked to complete the consent process. The consent process is described in great depth in that document. The interviewee had the option to leave at any time. Because these interviews took place during the height of the COVID-19 pandemic, they were all conducted remotely via Microsoft Teams, and each interview lasted approximately 30 – 60 minutes. Interviewees included mHealth project managers, mHealth development facilitators, and end users with extensive knowledge of the project. The interviews were digitally audio-recorded, transcribed, and thematically analysed. Thematic analysis is a method for examining and classifying patterns of meaning in a dataset.^44^ This involved the use of codes and themes to interpret and describe the phenomenon under study. The codes are created, and themes are generated from collating codes to get acquainted with data.^45^ Codes refer to an idea or feeling expressed in that part of a phrase in the data with the aim of organising data for subsequent interpretation, while themes refer to specific ideas and patterns of meaning that come up repeatedly from the codes, that captures something significant about the data or/and research questions.^46^ According to Braun and Clarke,^44^ thematic analysis ‘*provides a flexible and useful tool, which can potentially provide a rich and detailed, yet complex account of data’* (p.78). The analysis of the interview data is guided by the six-step model of thematic analysis outlined by Braun and Clarke.^44^

According to Maguire and Delahunt,^46^ ‘*thematic analysis is the process of identifying patterns or themes within qualitative data’ (p. 3352).* Since the six-step model of the thematic analysis described refers to themes, the notion of how the 12 themes were generated is explained in detail. The first step was to have a thorough overview of all the interview data about identifying what drives users to engage/disengage in the use/disuse of mHealth applications in order to design a better design process to improve user engagement in mHealth technologies. Hence, the concern is to address and analyse the data with the research question in mind. Initial codes were generated by highlighting phrases or sentences in the interview, and shorthand labels (codes) were devised to describe their content. Coding reduces the data into small chunks of meaning. The codes were developed and modified through the coding process without using pre-set codes. Coding was done using NVivo 12 (details in Appendix A), and the intention is not to code the content of the entire data set. The interview transcripts were coded separately by two persons (TI and PW). Each of the codes was compared, discussed, and modified before moving to the rest of the transcripts. Not every piece of the text was coded; however, each coded transcript was relevant to or specifically addressed the research question.

The next step was to examine the codes created and search for a theme with the analyses of the COM-B model – an idea or concept that captured and summarised the meaningful pattern and recurring pattern based on the analyses of the COM-B model. Braun and Clarke^44^ clarified that there are no certain rules about what makes a theme. A theme is described by its significance. At this stage, codes were examined to ensure that they fitted together into a broader theme in order to address the research question. The next step was to name, review, and refine the themes. Data associated with each theme were read to consider whether the data really did support the theme and how the themes work both within a single interview and across all the interviews. Naming themes involved providing a concise and easily understandable name for each theme. Themes were thus extracted from the transcripts of the interviews until we concluded that more themes could not be extracted from the data.

These interviews aimed at identifying what drives users to engage/disengage in the use/disuse of mHealth applications in order to design a better design process to improve user engagement in mHealth technologies. This obviously determined the interview questions and the analysis of the data. The interview participants were clear and consistent about what facilitates users’ engagement with mHealth technologies in the transcripts.

### COM-B model

The themes identified were analysed with the COM-B model^39,47^ to understand users’ behaviour of engaging with the mHealth app. By considering user engagement with an app as a behaviour, the COM-B model provides a broad framework for understanding mHealth application engagement. According to the COM-B model, behaviour (e.g. user engagement with app) arises from the interaction between the individual's capability, both physical (e.g. app skills) and psychological (e.g. knowledge of using an application), their opportunity to behave in a certain way, both physical (e.g. via features of the app) and social (e.g. resulting from recommendations to use an app), and their motivation to behave, both automatic (e.g. emotional rewards from using an app) and reflective (e.g. belief in the benefits of the app). Further details of the mapping process and the elements of the COM-B model are discussed in the following section.

## Results and discussions

**Figure 2.**
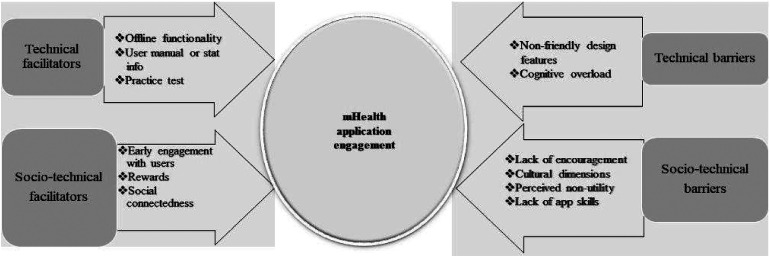
Classifying facilitators and barriers identified from the key findings into sociotechnical and technical factors.

The identified facilitators and barriers to mHealth application engagement in this case as derived from the interview data and mapped onto the components of the COM-B model are presented in Figure 1. These facilitators generated the following themes: involving user early in the design stages of the mHealth, providing social connectedness; offline functionality; practice test; user manual or guidance and statistical information; and tangible and intangible rewards. Each of these themes was classified as either sociotechnical or technical in order to determine whether the sociotechnical factors were deemed important for user engagement with mHealth in the transcript (as shown in Figure 2). In identifying facilitators to user engagement, participants also highlighted barriers to user engagement. These were grouped into the following themes: cognitive overload; non-user- friendly design; cultural dimensions; lack of encouragement; perceived non-utility; and lack of app skills. In the final step, each of these themes is classified as well, as either sociotechnical or technical in order to determine whether the sociotechnical factors were deemed important for user engagement with mHealth in this case (details in Appendix B).

The individual elements of the COM-B model and the outcome of the mapping process are now discussed in greater detail.

1. Physical capability refers to the appropriate skills and stamina that are required of users in order to take part in a particular behaviour. Many of the CHWs in this case struggle to engage with mHealth apps due to a lack of technical and language skills and low digital literacy. Several interviewees explained that CHWs expressed that the level of training provided to acquire technology skills shaped their level of commitment to engage with mHealth. This was highlighted by one interviewee as follows:
  *‘Ministry of Health and Sanitation in Sierra Leone provided training for CHWs to acquire skills to engage with mHealth apps in language of prefers, which helped to enhance learning uptake and engagement’ (D2)*. This indicates that CHWs could be helped to more fully engage with the mHealth app by offering app-use tutoring.
2. Psychological capability refers to a user's knowledge that is required to engage in a particular behaviour. All interviewees identified interface minimalism – i.e. making app interface as simple as it needs to be, as a factor that facilitates user engagement. Fewer elements on an interface result in lower cognitive load for users This factor is consistent with previous literature that reported engagement with health apps is impacted by factors affecting users’ capabilities that include different types of knowledge such as user guidance, statistical information, health information,^48,49^ and reduced cognitive load.^50–52^ The education level of users was highlighted by one respondent: *textit ‘When the MOTS mHealth solution was to be designed, the idea was to use smart phone but because of the level of education of users, basic features phones were recommended so that the CHWs can easily relate with’ (D3).* Furthermore, some of the interviewees reported that clear instructions on how to increase capability to perform a behaviour such as accessing the MOTS system by CHWs affects their engagement with an app. This is evidenced by the following quote: ‘*When a CHW delays in responding to instruction given by the system when accessed due to lack of clear instructions, call ends if phone key is not immediately pressed/navigated after instruction’ (D3).* This means that these CHWs could be provided with clear instructions on what they need to do to achieve a given task, to get fully engaged with the mHealth app.
3. Physical opportunity refers to the set of circumstances that make it easier for the users to engage with a behaviour. All the interviewees highlighted that an opportunity for two-way communication between the mHealth app and user, as well as user-friendly design and interaction, facilitates user engagement in this case. This aligns with the previous literature that explains how apps can be improved by targeting the design and engagement features, such as user-friendly design, or health professional support.^53,54^

Some interviewees suggested that careful selection of the terminology used to explain the app and what it does, such as simple and clear local language and pictorials, creates an impact on user engagement. ‘*The terminology used within the app, such pictorials, language, and aesthetic can affect user acceptability and engagement’ (D2).* In addition, the need for offline functionality was identified as a physical opportunity factor impacting user engagement. This was highlighted by the example provided by one of the interviewees about accessing the MOTS system from a weak or poor network connection, and how this negatively affects CHWs from engaging with it. In addition, an interviewee identified free network access and working with data in offline mode even when CHWs do not have access to the internet as an important factor for engagement: ‘*CHWs can use MOTS system without paying for it and work offline mode using Bluetooth technology to upload information to DHIS system’ (D1).*

This indicates that these CHWs could be assisted to engage with the mHealth app more fully by providing free internet and Bluetooth technology where there is no or poor network connection.

4. Social opportunity refers to a user's social circle enabling and facilitating a behaviour. Users’ sharing of knowledge and experiences of their mHealth app engagement within their social circle makes it easier for them to engage with mHealth app. The possibility for CHWs to share knowledge and experiences within mHealth apps was considered important social support that facilitates engagement with mHealth apps. According to,^55^ this type of social circle was found to improve intention to engage with a mobile app intervention designed for regular participation in physical activity during and after cancer treatment. In order to ensure that there is a social opportunity, we need to study users’ culture and capture it into mHealth apps. Here, culture is defined according to,^56^ as ‘*the patterns of thinking, feeling and acting that influence the way in which people communicate amongst themselves and with computers’ (p. 716)*.

Culture is divided into two layers – objective and subjective.^57^ Objective culture means that intended meaning of user interface representations, such as symbols, icons, and language, is translated to suit the target cultures so that they are understood correctly.^56^ While subjective culture ensures that interface representations reflect the values, ethics, and morals of the target users.^58^ This is evidenced by one particular comment from the interview as follows: ‘*Social and Cultural dimension needs to be incorporated in considerations of user engagement designs for user acceptability before deployment’ (D4).* This indicates that the user's social circle or community of practice, who also use the mHealth app, could assist with engagement. The social circle should be considered by integrating the social norms and cultures of users at the design and development stages of the mHealth app.

5. Automatic motivation refers to the user's reinforcement and emotions that sustain engagement with the mHealth app. All the interviewees stated that offering rewards and various non-financial incentives were found to be a useful way to increase engagement. This is also consistent with the literature,^59–61^ and this type of motivation is consistent with literature that observed that users found intangible rewards (e.g. badges) motivating,^62^ while others would want to receive tangible rewards instead (e.g. gift cards, cash, reduction in health insurance or vouchers provided by hospitals).^62,63^ Our interviewees confirmed this by stating the following about bonuses given to CHWs: ‘*At the end of the year or month CHWs that have performed very well are given bonuses which could be in a form of token payment, boots, or any other programmatic tools as a way of acknowledging them as outstanding users’ (D4).* This means that these CHWs could be provided with reinforcement and encouragement that will invoke their positive emotions to get them fully engage with the mHealth app.
6. Reflective motivation refers to users’ beliefs that their needs and values are reflected in the mHealth apps. Some of the interviewees observed that lack of perceived utility of the app during the design can hinder user engagement. Perceived utility refers to where there is no disparity between what the users’ needs and what an app offers. Siznay et al.^64^ observed that ‘*unmet expectations of an app would lead to disengagement and frustration with the app’ (p. 39).* This was particularly apparent in one interviewee's comment on CHW engagement with mHealth indicators: ‘*Users of mHealth apps such as CHWs may have issues engaging with mHealth apps if they are not involved in making sure that the indicators of their needs are reflected in the mobile apps, involving users to reflects their needs at the design stages of the mHealth apps, boosts their confidence of using mHealth apps’ (D2).* All the facilitators and barriers of engagement with mHealth interventions that were identified from the thematic analysis discussed above were classified as either technical or sociotechnical factors as shown in Figure 2. This was achieved using thematic analysis to identify the technical and sociotechnical facilitators and barriers to user engagement with mHealth in this case.

The presence of these technical and sociotechnical facilitators and barriers to user engagement with mHealth apps are important because they hold key positions for the mobile health system to sustain and scale as indicated by the COM-B analyses of data collected from interviewees. This aligns with the previous literature that discusses mHealth sustainability and scaling.^65–67^ Therefore, the presence of only techno-centric facilitators and barriers to user engagement with mHealth apps cannot offer improved user engagement. The facilitators and barriers identified, and the result of the COM-B analysis completed has informed the development of a new mHealth design framework called design process engagement enhancement system (DECENT). DECENT will allow designers/developers to uncover user engagement-impacting factors in order to improve user engagement. DECENT is discussed in detail in the following section. There are some limitations to this study. It was challenging to find information about pre/during/post-implementation of a specific mHealth. Although the use of criterion-based purposive sampling techniques minimised this effect, we could not guarantee that all potential participants would have been reached. Another limitation is that the information reported in this study reflects a single point-in-time measurement. We believe that a longitudinal study covering many time points combined with real-time user engagement and usage of mHealth apps would offer deeper insights into specific user views, particularly in the rapidly changing digital environment.

## Proposed design approach for user engagement – DECENT

**Figure 3.**
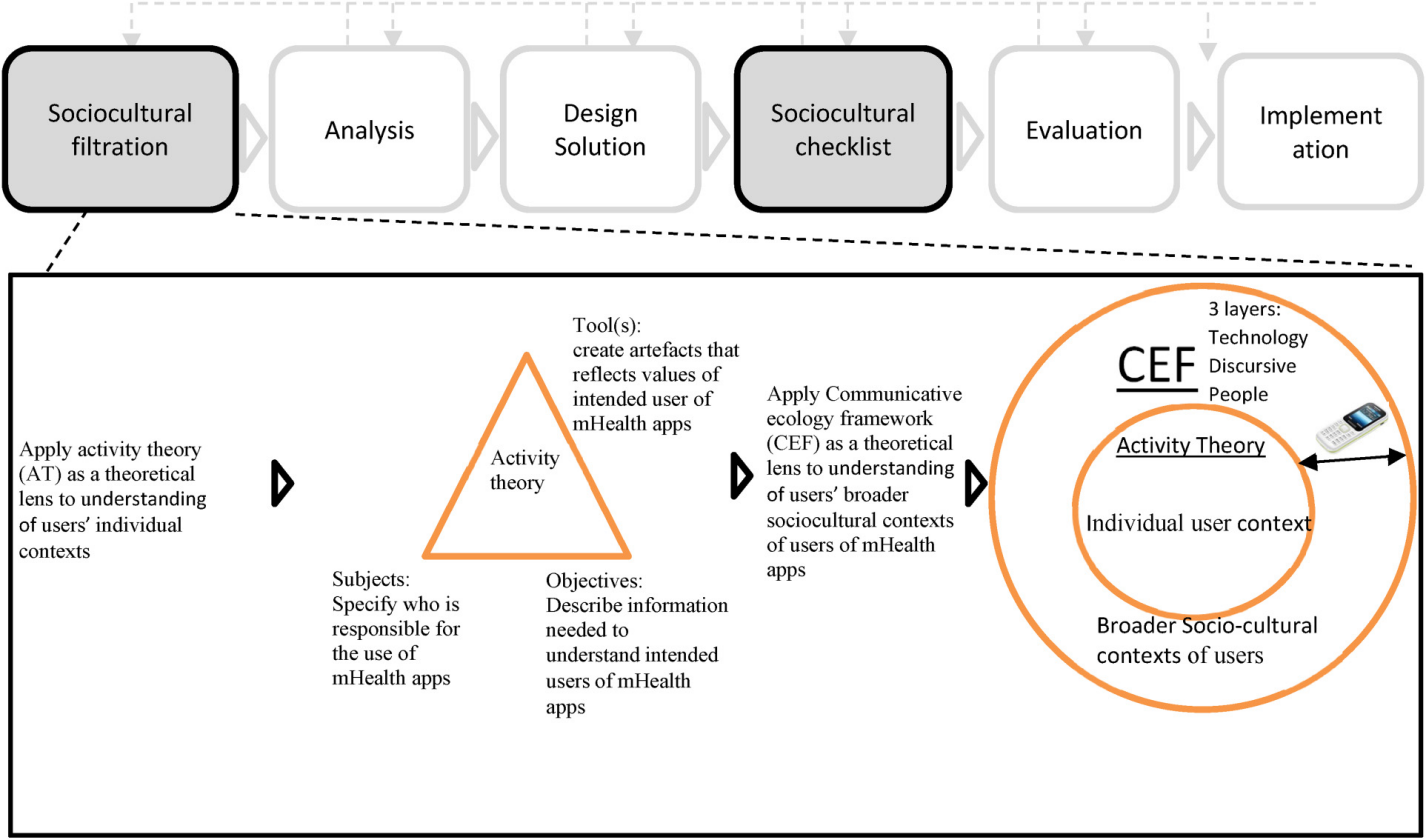
The design process engagement enhancement system (DECENT) model.

It is established in the results and discussion of this study that technical and sociotechnical facilitators as well as barriers to user engagement with mHealth apps are important because they hold key positions for mobile health systems to either succeed or fail. This study presents a new framework – DECENT – that will help mHealth designers and developers discover the key factors impacting user engagement with mHealth apps for end users and will serve as a methodological approach towards making decisions in the design, development, and implementation of mHealth for user engagement in the Global South. The proposed structure of DECENT is shown in Figure 3. The design science research is guided the design the DECENT framework.^52^ Figure 3 depicts the DECENT framework, which is divided into six phases. Phase 1: Social and cultural filtration presents use of AT and CEF theoretical lenses to describe the incorporation of individual as well as socio-cultural contexts of users into the mHealth design process, which aids mHealth designers and developers in the development of mHealth apps. User needs analysis, design solution development, a socio-cultural checklist, and evaluation, and implementation are also included in the DECENT framework. In the design of mHealth apps, DECENT prioritises aesthetic, socio-cultural, and contextual values, as well as a critical knowledge of the function of design in recognising and capturing users’ socio-cultural setting.^65^

The DECENT framework is a socio-culturally orientated process-focused user-centred design process. Figure 3 shows a socio-cultural filtration and checklist that fit readily into the first and second phases of an existing user-centred design framework. The socio-cultural filtration phase enables mHealth designers to understand the user's socio-cultural contexts; this is the input to the first user-centred design phase, the ‘analysis of user needs assessment’, which focuses on understanding the user's needs and values. Following the ‘design’ phase, the second phase of the user-centred design framework is a checklist. This checklist ensures that the solution addresses the socio-cultural features discovered during the first phase of socio-cultural filtration.^68^

DECENT incorporates various theories that will help designers and developers to uncover and take account of sociotechnical factors that impact user engagement with mHealth. One of the theories we propose to use is activity theory.^69^ Activity theory is a tool that can be used to uncover the sociotechnical context of mHealth implementations. We propose to leverage activity theory to support the creative design process and to provide a theoretical framework of social and cultural contexts in the design process of DECENT for user engagement.

Activity theory is embodied with primary units that aim towards incorporating social-technical content for user-centred design. The results of using the COM-B model (Figure 1) to analyse data and organise results show that CHWs struggle to engage with mHealth apps due to a lack of technical, language skills, and low digital literacy (i.e. Inner circle) in the COM-B model's physical capability component. Activity theory can be used to better understand the relationship between the literacy level of CHWs and mHealth apps in the language of preference in the uptake and use of mHealth apps. Similarly, using the COM-B model revealed that by providing app-use tutoring (i.e. facilitator- (outer)), CHWs could be assisted in engaging more fully with the mHealth app. The relationship between CHWs and app-use tutoring in the uptake and use of mHealth apps can be understood using activity theory.

Activity theory shows us the complexities and fluidity of activities in their immediate context and provides a strong framework for studying contextual factors. A significant development in the use of activity theory in research on how people use technology to solve new problems related to computers and information systems.^70,71^ Activity theory has always been particularly interested in how people interact with technology due to the theory's emphasis on tools and mediation. The theory enables a deeper comprehension of technology and its relevance to people interacting with technology in a cultural-historical personal context.^72,73^ To understand the broader socio-cultural contexts of the human relationship with technology, we complement activity theory with the communicative ecological framework that consists of three layers of interpretation (technical, social, and discursive) to provide a rich description of how people interact with technology.^74^ It is critical to understand not only the perspectives of mobile health developers and users but also the perspectives of all stakeholders involved in the activity system's activities and how they communicate. CEF is used to understand the relationships among stakeholders in the design of engaging apps and their communications mappings. CEF is composed of three layers^75^ as follows:

- The technology and media layer describes the methods used to communicate between various people and groups, and it includes all communication devices, distribution systems (whether digital or analog), and the technical systems that enable them (either software or mechanical).
- The discursive layer is ideational and focuses on communication content such as stories, understandings, beliefs, and symbols that define – in this case – design culture and design practices for user engagement.
- The people layer describes the various individuals and groups involved, as well as their social relationships and the social institutions and structures that link them.

A limitation of the mobile health framework as applied in this study was a low number of participants, as diversity and quantity of knowledge shared are beneficial to mobile health framework development. This limitation should be weighed with the time involved in engaging large groups to provide comprehensible knowledge of mobile health projects. Nevertheless, we have taken numerous steps to ensure confirmation of the integrity of the data.

The DECENT framework is intended to be used by mHealth designers and developers when developing mHealth apps/technologies in underserved settings, specifically in Sierra Leone. The framework is useful for design and development personnel who design mobile health applications for user engagement in Sierra Leone or Africa, as well as organisations such as World Vision Sierra Leone that implement mobile health projects such as MOTS to deliver refresher training to CHWs on the topic of vaccines and outbreak response.

As stated previously, DECENT prototypes were created based on the input from user-centred design models and the findings of semi-structured interviews. DECENT will be validated further by providing it to participants such as mHealth designers and end users, who will be able to explore their own ideas for something that might be valuable to them. DECENT will also apply a roadmap to future iterations of MOTs mHealth apps that are now in use in Sierra Leone's Bonthe areas.

## Conclusions

This work advances knowledge in the mHealth field by demonstrating that sociotechnical aspects of mHealth design are important for users in underserved settings, and it introduces the DECENT framework as a novel contribution of this study, which is designed from mHealth contexts in underserved settings in the Global South. However, it is not enough to simply analyse mHealth design from a sociotechnical perspective. Thus, this research proposes a new DECENT framework for designing mHealth for user engagement, which will be developed in order to aid inclusion of sociotechnical factors in mHealth. This will allow users’ needs and goals to be taken into account and advance identification of problems concerning lack of user engagement.

Although this research has provided promising results, part of our future work will discuss our proposed DECENT framework with a wide variety of stakeholders following the steps highlighted in this work. In addition, we will make a particular effort to engage as fully as possible with the CHWs in Sierra Leone as part of this process. This paper makes a contribution to the development of mHealth technologies by emphasising an understanding of the human-centred context and by putting forth related theoretical viewpoints that support technology development and end-user engagement. The foundation of sociotechnical research is the interdependence of any technological object with the social laws, customs, and practices, etc. that co-determine its creation, function, and use(s). The Global South has recently experienced unprecedented mHealth transformation as mHealth technologies have great potential in mediating and permeating human practices. Due to these quick changes, achieving end-user engagement with technological solutions has become extremely difficult. This paper emphasises the significance of recognising and identifying the sociotechnical perspective of mHealth technology, in a Sierra Leone case setting. We can draw the conclusion that our approach supports the credibility, transferability, confirmability, and dependability of the analysis, which accurately reflects the perspectives shared by the group of participants across various Sierra Leonean locations.^76,77^

## Appendix A

Coding

## Appendix B

Extracting themes

### Interview questions

1. What is your name? What is your organisation and title?
2. Can you tell me about your role?
3. What experience do you have working with mobile Health?
4. Can you tell me about any specific mHealth project or MOTS that you are currently involved with? Note: include previous mHealth projects.
5. What does ‘user engagement’ mean to you?
6. Do you think user engagement is important?
7. At what point is UE important? (designing, developing, and implementing mHealth) Prompt: ask about user engagement in the three stages-design, development and implementation.
8. Do you think users are engaged with this project and the mHealth app? Please explain this answer if possible.
9. Do you monitor the way mHealth app users have engaged with the app? If so, how? If not, why not?
10. Are you aware of any formal models, frameworks or processes to do this? Have you ever used any of these models/processes? If not, how do you do this?
11. Have you received any other feedback from users about the design of the mHealth apps?
12. Were there any attempts to increase/improve user engagement in this mHealth project? If so, please explain.
13. Were there any facilitators/barriers to user engagement in this project?
14. How were the facilitators exploited it or used?
15. How were the barriers overcome?
16. Are there any sociotechnical facilitators/barriers to UE? Prompt – CHWs and incentives and payment for their work.
17. How can we increase/improve/maximise user engagement in mHealth? Prompt for a longer and more detailed answer if needed.
18. Could you please explain how the experience of using the app serves users’ needs and how it could influence user engagement?
19. Is there anything else you would like to tell me that I have not asked?
